# Dual-energy X-ray absorptiometry measures of lean body mass as a biomarker for progression in boys with Duchenne muscular dystrophy

**DOI:** 10.1038/s41598-022-23072-5

**Published:** 2022-11-05

**Authors:** Sarah P. Sherlock, Jeffrey Palmer, Kathryn R. Wagner, Hoda Z. Abdel-Hamid, Cuixia Tian, Jean K. Mah, Francesco Muntoni, Michela Guglieri, Russell J. Butterfield, Lawrence Charnas, Shannon Marraffino

**Affiliations:** 1 Pfizer Inc, Cambridge, MA USA; 2 Kennedy Krieger Institute, Johns Hopkins School of Medicine, Baltimore, MD USA; 3 Division of Child Neurology, Department of Pediatrics, UPMC Children’s Hospital of Pittsburgh, University of Pittsburgh, Pittsburgh, PA USA; 4 Cincinnati Children’s Hospital Medical Center, Cincinnati, OH USA; 5 University of Cincinnati School of Medicine, Cincinnati, OH USA; 6 Alberta Children’s Hospital, Cumming School of Medicine, University of Calgary, Calgary, AB Canada; 7 Dubowitz Neuromuscular Centre, NIHR Great Ormond Street Hospital Biomedical Research Centre, Great Ormond Street Institute of Child Health, University College London, London, UK; 8 John Walton Muscular Dystrophy Research Centre, Translational and Clinical Research Institute, Newcastle University and Newcastle Hospitals NHS Foundation Trust, Newcastle, UK; 9 University of Utah School of Medicine, Salt Lake City, UT USA

**Keywords:** Neuromuscular disease, Predictive markers

## Abstract

We evaluated whether whole-body dual-energy X-ray absorptiometry (DXA) measures of lean body mass can be used as biomarkers for disease progression and treatment effects in patients with Duchenne muscular dystrophy. This post hoc analysis utilized data from a randomized, 2-period study of domagrozumab versus placebo in 120 ambulatory boys with DMD. DXA measures of lean body mass were obtained from the whole body (excluding head), arms, legs and appendicular skeleton at baseline and every 16 weeks. Treatment effects on DXA measures for domagrozumab versus placebo were assessed at Week 49. At Week 49, domagrozumab statistically significantly increased lean body mass versus placebo in the appendicular skeleton (*p* = 0.050) and arms (*p* < 0.001). The relationship between lean body mass at Week 49 and functional endpoints at Week 97 was evaluated. Changes in lean body mass at Week 49 in all regions except arms were significantly correlated with percent change from baseline in 4-stair climb (4SC) at Week 97. DXA-derived percent lean mass at Week 49 also correlated with 4SC and North Star Ambulatory Assessment at Week 97. These data indicate that whole-body DXA measures can be used as biomarkers for treatment effects and disease progression in patients with DMD, and warrant further investigation.

*Trial registration*: ClinicalTrials.gov, NCT02310763; registered 8 December 2014.

## Introduction

Duchenne muscular dystrophy (DMD) is a recessive, X-linked genetic disorder caused by mutations in the *DMD* gene, which leads to deficiency of the protein dystrophin^1^. In patients with DMD, the progressive loss of myocytes in skeletal muscle leads to replacement by adipose and fibrous tissue, with the majority of patients experiencing loss of ambulation in early teenage years, and premature death due to respiratory and/or cardiac failure in early adulthood^2,3^. Treatment of DMD requires a coordinated multidisciplinary approach; approved treatments act to ameliorate symptoms and include glucocorticoids, as well as more recent nonsense mutation readthrough and exon skipping therapies^4–9^. Multiple therapeutic strategies are under investigation for patients with DMD; however, demonstrating the effect of therapeutic interventions may be challenging due to the clinical heterogeneity among patients, including response to concomitant therapies, type of mutation, adherence and other confounding factors (e.g. motor and cognitive development). In addition, lack of consistent functional outcome measures across different ages or disease stage, combined with the variable time for changes to become apparent, make it difficult to detect small yet potentially meaningful changes in response to treatment^10,11^. Hence, there is a need for robust biomarkers with objective measures that can monitor disease progression and predict treatment effects across a range of possible treatment-effect sizes.

Whole-body dual-energy X-ray absorptiometry (DXA) is a sensitive and accurate method for quantifying body composition, including both fat mass and lean body mass as a surrogate measure of skeletal muscle^12^. DXA is useful in the assessment of body composition secondary to endocrine disorders and in pediatric patients with delayed growth^12^. DXA has also been used to assess fat mass and lean body mass in patients with DMD, demonstrating that DXA measures of lean body mass correlate with function and strength^13,14^, and is also routinely used to assess bone health in patients with DMD. Patients with DMD receiving glucocorticoids consistently had lower DXA-measured appendicular lean body mass compared with age-matched healthy controls^15^. A separate study showed DXA-measured increases in lean body mass in patients with DMD following 2 years of steroid treatment compared with steroid-naïve patients, with no concomitant changes in body fat^16^. Whether DXA measures of lean body mass can be used as a robust biomarker for disease progression and treatment effects in patients with DMD has yet to be explored.

Domagrozumab is a humanized recombinant monoclonal immunoglobulin antibody subclass 1 (IgG1) that neutralizes myostatin (GDF-8)^17,18^. Since myostatin is a negative regulator of skeletal muscle mass, an anti-myostatin therapy would be predicted to increase muscle mass and potentially modify the natural history of DMD^19^. The therapeutic goal for domagrozumab is to slow disease progression in DMD. Preclinical studies of domagrozumab in non-human primates demonstrated dose-dependent increases in lean body mass, as assessed by DXA, and muscle volume, as assessed by computerized tomography scan^20^. In healthy volunteers, domagrozumab was well tolerated and associated with a 5.4% increase in whole-body lean body mass (10 mg/kg single-dose cohort, as measured by DXA) and 4.5% increase in muscle volume (3 × 10 mg/kg repeat-dose cohort, as measured by magnetic resonance imaging [MRI])^21^. Domagrozumab was evaluated in a phase 2 multicenter clinical trial as a potential therapy for participants with DMD (NCT02310763)^17,18^. The phase 2 study, in addition to safety and functional endpoints, characterized changes in MRI-derived measures of muscle and DXA-derived measures of lean body mass as secondary and exploratory biomarkers in DMD. The muscle MRI analysis revealed a significant difference between domagrozumab and placebo in the mean percent change from baseline (%CFB) in thigh muscle volume at Week 49 (difference of 4.09%, *p* = 0.030)^17,18,22^. The aims of this post hoc analysis of data collected during the phase 2 study were to assess the longitudinal DXA measurement results in response to treatment with domagrozumab and to understand the potential of DXA lean body mass measures as a predictive biomarker for functional improvement.

## Results

### Participants

Of 121 randomized participants, 120 received treatment with domagrozumab or placebo and all participants were analyzed for primary efficacy measures and whole-body DXA. Baseline participant characteristics were comparable between domagrozumab and placebo treatment groups (Table 1).

**Table 1:** Baseline participant characteristics and DXA measures in the overall population.

Participant characteristics	Domagrozumab, n = 80	Placebo, n = 40	Overall, N = 120
Age, y (SD) Range	8.4 (1.7) 6–14	9.3 (2.3) 6–15	8.7 (2.0) 6–15
Weight, kg (SD) Range	30.1 (8.6) 14.8–50.1	35.3 (14.4) 19.0–86.4	31.8 (11.1) 14.8–86.4
BMI^a^, kg/m^2^ (SD) Range	19.4 (3.8) 11.7–29.4	20.7 (5.8) 13.3–39.7	19.9 (4.6) 11.7–39.7
Number of participants with DXA measures, n	77	40	117
Whole body: Lean body mass, kg (SD)	15.6 (3.2)	17.6 (4.4)	16.2 (3.8)
Whole body: Fat mass, kg (SD)	9.9 (5.5)	12.8 (9.7)	10.9 (7.3)
Appendicular skeleton: Lean body mass, kg (SD)	7.0 (1.7)	8.0 (2.5)	7.3 (2.1)
Appendicular skeleton: Fat mass, kg (SD)	5.5 (2.9)	6.7 (4.5)	5.9 (3.6)
Total leg: Lean body mass, kg (SD)	5.2 (1.3)	6.0 (1.9)	5.5 (1.6)
Total leg: Fat mass, kg (SD)	4.2 (2.3)	5.2 (3.5)	4.6 (2.8)
Total arm: Lean body mass, kg (SD)	1.7 (0.4)	2.0 (0.6)	1.8 (0.5)
Total arm: Fat mass, kg (SD)	1.2 (0.7)	1.5 (1.0)	1.3 (0.8)
Percent lean mass, kg (SD)	63.5 (10.4)	61.7 (11.3)	63.0 (10.7)

All values represent the mean unless otherwise stated.

^a^BMI was defined as weight/(height × 0.01)^2^.

*BMI* Body mass index; *DXA* Dual-energy X-ray absorptiometry; *SD* Standard deviation.

### DXA analysis

**Figure 1:**
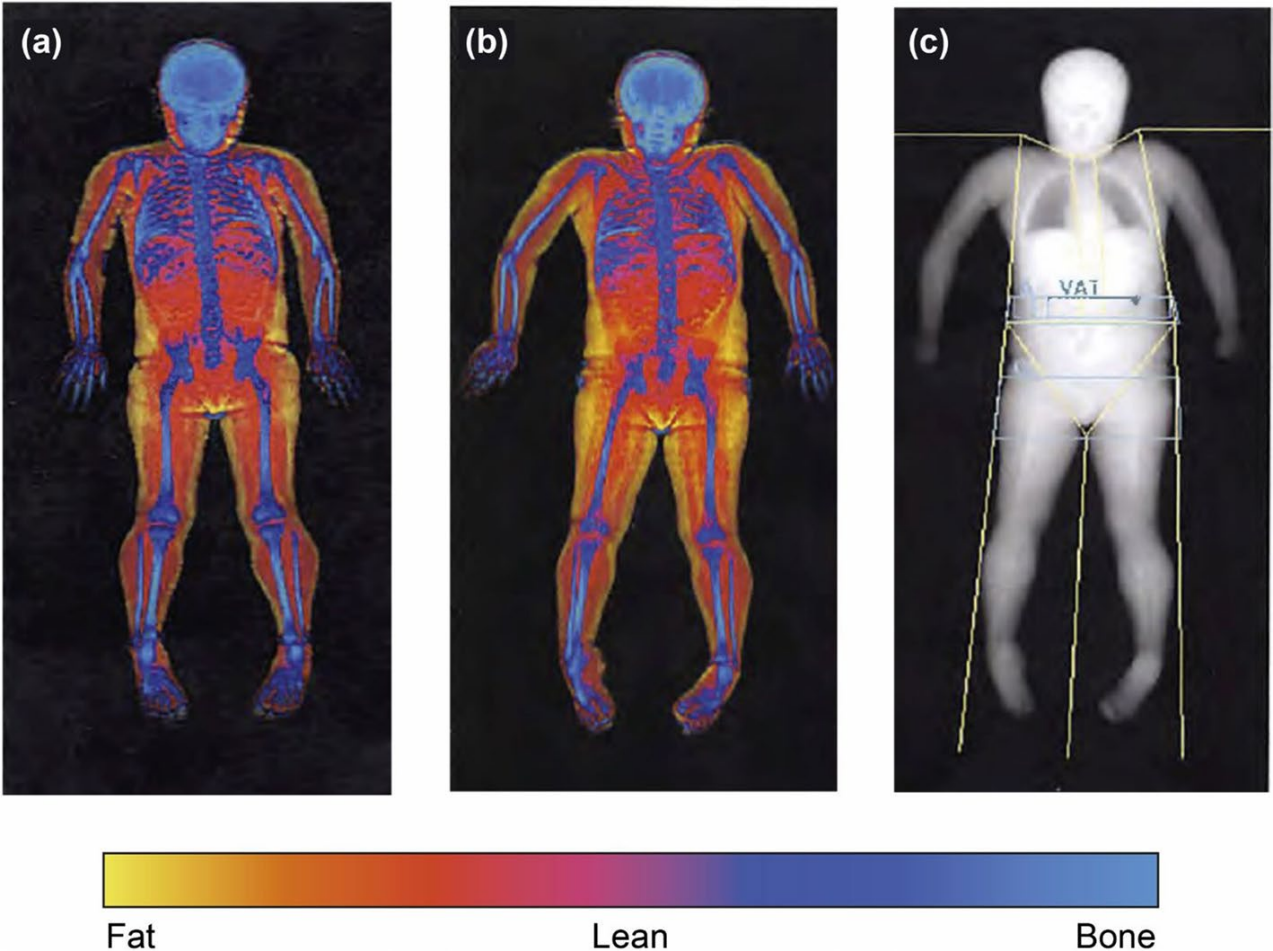
Representative DXA scan. A visual representation of fat mass, lean body mass and bone mass is shown for a participant (**a**) before treatment and (**b**) at the Week 49 visit. Cutlines are placed on the DXA scan to separate regions of interest for analysis (**c**). Primary regions for analysis included arms (left and right combined), legs (left and right combined), appendicular skeleton (arms and leg as a single region of interest) and total body excluding the head. Cutlines were placed by a trained analyst and reviewed by a radiologist prior to finalizing read results. DXA, Dual-energy X-ray absorptiometry.

**Figure 2:**
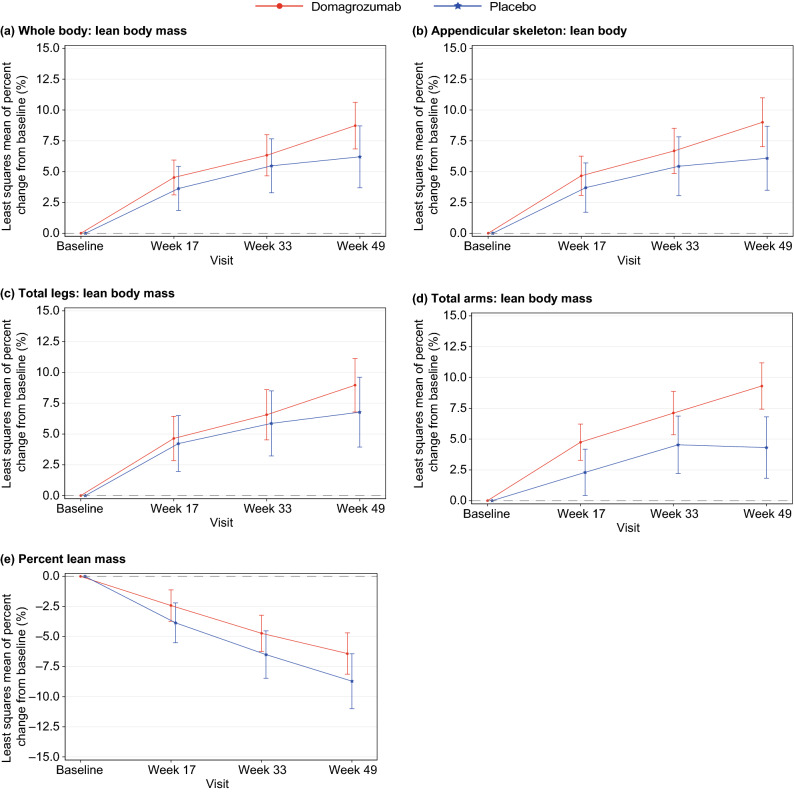
Change from baseline in DXA lean body mass. Least-squares mean change from baseline in lean body mass at Weeks 17, 33 and 49 for (**a**) whole body, (**b**) appendicular skeleton, (**c**) total legs and (**d**) total arms; (**e**) shows percent lean mass. DXA, Dual-energy X-ray absorptiometry.

A representative participant’s DXA scan is shown in Fig. 1. Fat mass, lean body mass, and bone mass are shown pre-dose (Fig. 1a) and at the Week 49 visit (Fig. 1b). Regions of interest are separated by cutlines for analysis (Fig. 1c). DXA results of lean body mass changes in different regions of interest at Weeks 17, 33 and 49 are shown in Fig. 2.

Compared with placebo, treatment with domagrozumab was associated with a nominal increase in lean body mass in all regions of interest, while slowing the decline in whole-body percent lean mass (Fig. 2). Compared with placebo at Week 49, statistically significant increases in lean body mass were observed in the appendicular skeleton (mean difference 2.9, 95% CI − 0.02, 5.87; *p* = 0.050) and arms (mean difference 5.0, 95% CI 2.16, 7.84; *p* < 0.001), with a trend toward an increase in the whole body (mean difference 2.5, 95% CI − 0.35, 5.41; *p* = 0.080; Table 2, Fig. 2). Similarly, percent lean mass appeared to be preserved in domagrozumab-treated participants versus placebo; however, this was not statistically significant (mean difference 1.9, 95% CI − 0.78, 4.65; *p* = 0.16).

**Table 2:** Change from baseline in lean body mass and fat mass after 48 weeks of treatment.

DXA measurement		Treatment	Adjusted mean (95% CI)	Difference (95% CI)	*p* value
Whole-body	Lean body mass	Domagrozumab	8.7 (6.82, 10.58)	2.5 (− 0.35, 5.41)	0.08
		Placebo	6.2 (3.71, 8.69)		
	Fat mass	Domagrozumab	30.5 (24.40, 36.60)	− 4.6 (− 14.34, 5.07)	0.35
		Placebo	35.1 (26.85, 43.35)		
Appendicular skeleton	Lean body mass	Domagrozumab	9.0 (7.04, 10.96)	2.9 (− 0.02, 5.87)	0.050
		Placebo	6.1 (3.53, 8.67)		
	Fat mass	Domagrozumab	28.5 (22.58, 34.42)	− 4.7 (− 14.15, 4.82)	0.33
		Placebo	33.1 (25.06, 41.14)		
Legs	Lean body mass	Domagrozumab	9.0 (6.84, 11.16)	2.2 (− 1.00, 5.38)	0.18
		Placebo	6.8 (4.00, 9.60)		
	Fat mass	Domagrozumab	29.0 (23.18, 34.82)	− 3.6 (− 12.93, 5.78)	0.45
		Placebo	32.6 (24.68, 40.52)		
Arms	Lean body mass	Domagrozumab	9.3 (7.44, 11.16)	5.0 (2.16, 7.84)	< 0.001
		Placebo	4.3 (1.83, 6.77)		
	Fat mass	Domagrozumab	27.7 (20.94, 34.46)	− 8.7 (− 19.48, 2.05)	0.11
		Placebo	36.4 (27.25, 45.55)		
Percent lean mass		Domagrozumab	− 6.0 (− 7.59, − 4.49)	1.9 (− 0.78, 4.65)	0.16
		Placebo	− 8.0 (− 10.15, − 5.79)		

Data are % change from baseline. All results were calculated using a mixed model of repeated measures.

*CI* Confidence interval; *DXA* Dual-energy X-ray absorptiometry.

No statistically significant difference in fat mass between domagrozumab and placebo groups was observed in any region of interest (Table 2). Compared with placebo, the effects of domagrozumab appeared to become more pronounced after Week 33 in all DXA measures of lean body mass (Fig. 2). In the placebo group, there was a consistent gradual decline in percent lean mass, whereas lean body mass measures in the arms, legs, appendicular skeleton and whole body appeared to be increasing up until Week 33, after which these measures generally remained stable (Fig. 2).

### Correlative analysis of DXA measures with 4SC time and NSAA score

Percent CFB in DXA measures of lean body mass at Week 49 were compared by linear regression against the %CFB in 4-stair climb (4SC) time and CFB in North Star Ambulatory Assessment (NSAA) at Week 97 to assess whether changes in lean body mass may precede functional changes. All changes in lean body mass at Week 49 except for arms (*p* = 0.23) were significantly correlated with the %CFB in 4SC time at Week 97 (Table 3). Participants who had a large percent increase in whole-body lean body mass at Week 49 (> 4.2%CFB) performed better on 4SC at Week 97 (33.1 %CFB) compared with participants who had a small percent change in whole-body lean body mass (100.6 %CFB). Regression tree analyses on all other DXA endpoints yielded a difference in percent change in 4SC between the subgroups of a similar magnitude, except for lean body mass as measured in arms.

**Table 3:**
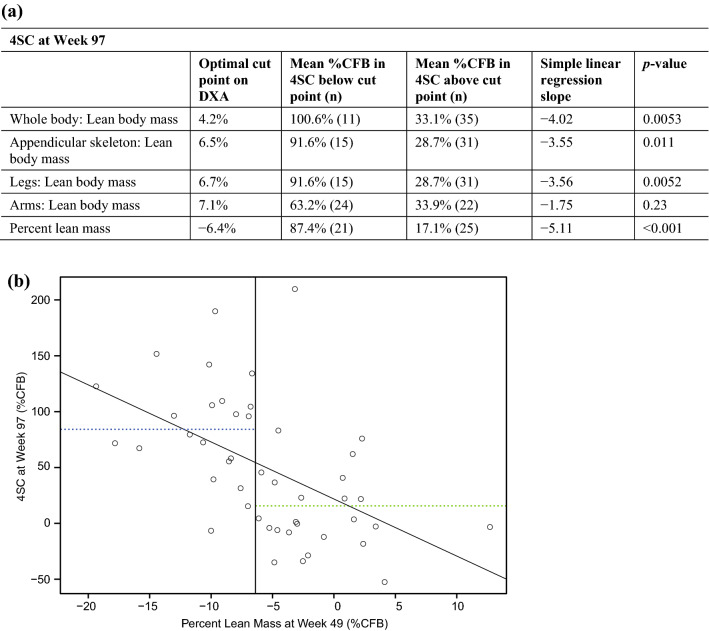
Comparison of DXA change from baseline at Week 49 versus 4SC at Week 97.

(**a**) Linear regression analysis of DXA %CFB at Week 49 versus 4SC at Week 97 in the overall population, and (**b**) example regression scatter plot showing the relationship between percent lean mass at Week 49 and 4SC at Week 97. In (**b**), the vertical line is the optimal cut point, the blue dotted line is the average response below the cut point, the green dotted line is the average response above the cut point, and the diagonal line is the simple linear regression fit. %CFB, percent change from baseline; 4SC, 4-stair climb; DXA, dual-energy X-ray absorptiometry; n, number in subgroup.

Only changes in percent lean mass at Week 49 were significantly correlated with changes from baseline in NSAA scores at Week 97 (*p* < 0.001; Table 4). Patients who had a higher percent change in percent lean mass at Week 49 (> 5.5 %CFB) performed substantially better on NSAA (− 2.0 CFB) compared with participants who had a lower percent change in percent lean mass (− 9.1 CFB), a difference of > 7 points on the NSAA scale.

**Table 4:**
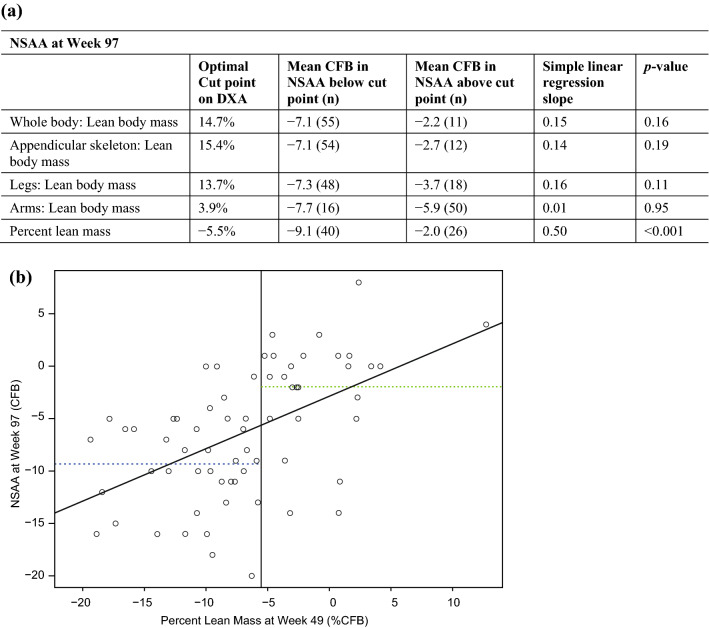
Comparison of DXA change from baseline at Week 49 versus NSAA at Week 97.

(**a**) Linear regression analysis of DXA %CFB at Week 49 versus NSAA at Week 97 in the overall population, and (**b**) example regression scatter plot showing the relationship between percent lean mass at Week 49 and NSAA at Week 97. In (**b**), the vertical line is the optimal cut point, the blue dotted line is the average response below the cut point, the green dotted line is the average response above the cut point, and the diagonal line is the simple linear regression fit. %CFB, percent change from baseline; CFB, change from baseline; DXA, dual-energy X-ray absorptiometry; n, number in subgroup; NSAA, North Star Ambulatory Assessment.

## Discussion

Treatment with domagrozumab compared with placebo was associated with statistically significant increases in lean body mass in the appendicular skeleton and arms compared with placebo, as well as significant increases in thigh muscle volume as measured by MRI^17^. However, the phase 2 study did not meet its primary efficacy endpoint (mean CFB in 4SC after 1 year of domagrozumab treatment vs. placebo)^17^. Evaluation of the totality of evidence did not support a significant treatment effect and the decision was made to terminate the study. Despite promising preclinical data, there has been little success with the development of myostatin inhibitors for the treatment of DMD, including domagrozumab, ACE-031 and others^23^. Potential explanations are reviewed fully elsewhere^17,23^, but include differences in native myostatin levels in mice compared with humans, variation in the suppression of circulating myostatin between healthy and DMD muscles and confounding effects of corticosteroid treatment. Nevertheless, our analysis provides evidence to support the pharmacodynamic effect of myostatin inhibition in patients with DMD as measured by DXA, which, unfortunately, was not sufficient to translate into consistent functional improvements.

Our analysis of data from the phase 2 domagrozumab trial supports the use of DXA measures of lean body mass as a biomarker in monitoring the therapeutic effects of a pharmacological intervention in patients with DMD. After 48 weeks of domagrozumab treatment, a nominal increase in lean body mass was observed in all regions of interest, with statistically significant increases in the appendicular skeleton and arms as compared with placebo. The increase in lean body mass observed in the appendicular skeleton was likely driven by the large increase in the arms, as the appendicular skeleton region of interest includes both arms and legs into a single region of interest. The increase in lean body mass observed in the arms and appendicular skeleton of boys treated with domagrozumab could be attributed to the amount of healthy muscle remaining in the arms relative to the legs in boys with DMD, as legs are affected more severely and earlier than arms in patients with DMD^24,25^. The larger amount of healthy muscle in the arms may provide increased substrate for myostatin inhibition. In addition, the lack of significant effect on fat mass measures over 49 weeks is consistent with the expected mechanism of action of myostatin inhibition.

Linear regression measures were conducted to understand the relationship between shorter-term changes in DXA measures of lean body mass (49 weeks) and longer-term functional changes (97 weeks). This post hoc analysis was done to understand if DXA measures could help predict functional decline in future clinical studies. Almost all lean body mass measures, except for arms, directly correlated with functional assessments, including 4SC time at Week 97. In addition to assessing the correlations between Week 49 lean body mass changes and Week 97 functional changes, optimal cut points, which separated each biomarker into two subgroups, were identified using regression tree methods. The optimal cut point provides a threshold for the given biomarker that maximizes the difference between functional outcomes after 97 weeks. The optimal cut point in Week 49 DXA measures of lean body mass were all between − 10% and 10%, suggesting that even small shifts in body composition could be meaningful on longer-term function. Maintenance or increases in lean body mass led to more favorable 4SC performance after 97 weeks. Percent lean mass was also significantly correlated with 4SC times, with an optimal cut point of − 6.4%. This could be interpreted as boys who had better maintenance of lean body mass, relative to fat mass accumulation, performing better on the 4SC after 97 weeks.

Linear regression analysis and the use of optimal cut points were also performed looking at NSAA measures after 97 weeks. In this analysis, only percent lean mass was significantly correlated with NSAA changes after Week 97, with an optimal cut point of − 5.5%. This strong correlation, and difference in NSAA scores above and below the optimal cut point, further reinforces the importance of looking at lean body mass relative to total tissue mass in future DXA studies. The lack of correlation between DXA lean body mass measures with NSAA changes after 97 weeks was unexpected, although could be an important consideration when selecting DXA measures in future DMD studies. Overall, the linear regression analysis suggests that percent lean mass measure may be the strongest predictor of future functional changes. However, this finding would need to be validated in future studies.

Domagrozumab appeared to have a favorable effect on lean body mass in the arms and appendicular skeleton. However, there was no significant effect on whole-body lean body mass or percent lean mass relative to placebo. This lack of effect may help to explain why there were no significant differences on function as a result of treatment with domagrozumab^17^. In addition, despite the favorable increase in lean body mass in the arms, there were no treatment-related effects on the performance of upper limb assessment observed during the 48-week treatment period, suggesting that the small increases in lean body mass in the arms may not have been sufficient to induce a functional change^17^.

There is an unmet need for accurate, affordable and reliable biomarkers to monitor DMD progression and evaluate the efficacy of therapeutic interventions. Although numerous types of DMD-related biomarkers to monitor therapeutic response and pharmacodynamics have been identified, they are not routinely employed in everyday practice and in most cases require further validation to establish correlation with clinically specific milestones^26^. DXA scans are routinely used in patients with DMD to assess bone health^2^ and could be used in future DMD studies to evaluate trial drug effects on lean body mass to assess disease progression. Furthermore, DXA provides an objective and rapid method of monitoring changes in body composition, including both regional and whole-body estimates^12^. DXA scans are well tolerated by young patients due to the open-table set-up and short scan times (< 10 min). Inclusion of a whole-body scan into this annual examination would present minimal additional burden for the patient. Furthermore, several studies have demonstrated a relationship between DXA-derived lean body mass measures and muscle strength in patients with DMD, in addition to demonstrating that males with DMD have lower lean body mass as compared with heathy males^13–15,27–29^.

DXA scans have several limitations that are important to consider in DMD disease progression. DXA provides an estimate of bone mass, lean body mass and fat mass, based on the differential flux of X-rays from these three body compartments. Soft tissues (lean body mass and fat mass) are mostly composed of water and organic compounds^30^, therefore fluid retention from any cause can affect lean body mass measurements^31^. Accumulation of fibrous tissue in boys with DMD can also affect lean body mass measures because DXA does not differentiate between healthy muscle and fibrous tissue and, therefore, measures of functional muscle mass could be overestimated. As DMD progresses, the percentage of total muscle volume decreases as muscle is replaced by fat and fibrous tissue^32^. DXA does not differentiate fat infiltration from intermuscular or subcutaneous fat, and therefore cannot provide a completely accurate interpretation of fat distribution. In some cases, increases in fat mass measures could be a function of background therapy with glucocorticoids rather than a true representation of disease progression. In DMD studies, MRI is often considered as a potential biomarker due to the strength in differentiating fat types and distribution^33^.

Despite any shortcomings, DXA has been successfully utilized in several clinical studies to assess body composition and provide important insights into the action of therapeutic agents. In the phase 2 study of the myostatin inhibitor ACE-031, ambulatory boys with DMD experienced nominal increases in lean mass %CFB at Day 99 in both ACE-031 cohorts (+ 3.6% and + 4.1%; *p* < 0.05 for each) and placebo (+ 2.6%)^34^. The %CFB in body fat mass to Day 99 appeared to favor boys with DMD who received ACE-031 (Cohort 1, − 0.9%; Cohort 2, + 3.4%) compared with placebo (+ 6.8%), although statistical significance was not reached^34^. In the phase 2b trial of bimagrumab in adults aged 36–85 years with inclusion body myositis, there was a dose-dependent increase in lean body mass after 52 weeks as measured by DXA^35^. The modest increase in lean body mass observed with bimagrumab did not translate to functional improvements (as measured by CFB in 6-min walk distance) but did confirm its biological activity in skeletal muscle^35^. Notably, the pharmacodynamic effects of both ACE-031 and bimagrumab include actions on lean body mass and fat mass, complicating interpretation of these findings and highlighting the difficulty in correlating pharmacodynamic drug effects and body composition changes. In a comparison of 23 patients with DMD and 23 control subjects, DXA measurements of body composition demonstrated that patients with DMD have decreased regional lean mass, increased regional fat mass and decreased strength compared with control subjects^14^. In a separate study, patients with DMD who received 2 years of treatment with prednisone and/or deflazacort had a decrease in the rate of decline in Motor Function Measure, which was associated with increases in lean tissue mass as measured by DXA^16^.

Although this work continues to support DXA measures of lean body mass as a potential biomarker in DMD, this study has limitations. All DXA endpoints were exploratory and were therefore not the sole focus of this work. The study was designed as an interventional study and was not intended to test the utility of DXA measures as a biomarker to predict future functional changes. The relationship between DXA measures and future functional outcomes could have been affected by changes in therapeutic regimens in the second period of this study^17^. However, results for each of the three treatment sequences in the study were similar to the pooled results. This study was terminated early for lack of clear efficacy and therefore not all patients were followed until their Week 97 functional assessment, posing a limitation in interpreting the correlative relationship between DXA measures at Week 49 and long-term functional change. In addition, functional outcome measures, including the 4SC and NSAA, are subject to multiple co-variates among patients, including ankle contractures, patient motivation, age and mutations that may have influenced the functional outcomes. Therefore, the correlation of DXA measurements and functional measures will require further validation in clinical trials.

In conclusion, whole-body DXA is a sensitive method that can be successfully implemented in multicenter clinical studies to detect therapeutic drug effects on body composition in patients with DMD. Following 48 weeks domagrozumab treatment there was a nominal increase in lean body mass in all regions of interest, with statistically significant increases in the appendicular skeleton and arms. Correlation between functional measures, the 4SC and NSAA, and lean body mass as measured by DXA suggest that DXA changes could precede functional changes and warrant further investigation and validation in clinical trials.

## Methods

### Study design

This analysis was based on data collected during the randomized, 2-period (48 weeks each, 96 weeks total), double-blind, placebo-controlled, intra-participant, multiple ascending-dose study of domagrozumab in ambulatory boys with DMD aged 6 to < 16 years^17^. The trial was conducted in accordance with legal and regulatory requirements, as well as the general principles set forth in the International Ethical Guidelines for Biomedical Research Involving Human Subjects, guidelines for Good Clinical Practice and the Declaration of Helsinki. The protocols, any amendments and informed consent/assent documents were approved by the institutional review board or ethics committee at each study center (a full list of institutional review boards and ethics committees for participating centers can be found in Supplementary Table S1). Parents or legal guardians provided written, informed consent prior to any study-specific activity being performed.

Beginning in November 2014, 31 sites in eight countries (USA, Canada, UK, Japan, Italy, Bulgaria, Poland and Australia) screened 162 boys; 121 were enrolled and 120 treated. Primary safety and efficacy analyses were completed when all enrolled participants completed 48 weeks treatment (Period 1) in June 2018 and have been reported^17,18^. Eligible participants were able to complete the 4SC in ≥ 2.5 and < 12 s at screening and were on a stable dose of glucocorticoid steroids at ≥ 6 months^17^.

The primary objectives of the phase 2 study were to (i) determine the safety and tolerability of multiple ascending repeat intravenous doses of domagrozumab in ambulatory boys with DMD, and (ii) demonstrate the efficacy of treatment based on an observed mean CFB on function (4SC) as compared with placebo following 49 weeks of treatment. Participants were randomized in a 1:1:1 ratio into one of three sequence groups. Three domagrozumab dose levels (5, 20, and 40 mg/kg) were investigated in a within-participant dose-escalating fashion. Dosing occurred every 4 weeks (28 days)^17^. In Sequence 1, domagrozumab within-participant dose escalation was followed by 40 mg/kg (tolerated by all boys) for Weeks 49–96. In Sequence 2, domagrozumab within-participant dose escalation in the first 48 weeks was followed by placebo (Weeks 49–96); and in Sequence 3, placebo was followed by domagrozumab within-participant dose escalation (Weeks 49–96)^17^. A schematic of the study design can be found in the original article reporting the findings of this trial^17^. Efficacy was evaluated at Week 49 by comparing a single active group (Sequences 1 + 2) with placebo (Sequence 3). Lean body mass as measured by DXA was an exploratory study objective, and correlation between lean body mass and function were analyzed post hoc.

### DXA and image analysis

Participants were asked to avoid large meals for at least 2 h prior to the DXA scan but were to be in a state of euhydration to minimize variation in lean mass measures due to varying hydration levels^36^. All images were collected from imaging facilities trained on the specific requirements of the study. Site staff were trained on participant preparation, participant positioning, image quality control and essential quality assurance requirements for each scanner. DXA scanning followed standard clinical scan acquisition protocols. Sites were instructed to follow the same imaging protocols at all time points and use consistent participant positioning techniques. Following scan acquisition, DXA scans were transferred to an independent central radiology imaging facility (Biotelemetry Research, Rochester, NY) for quality control and central analysis.

Central quality inspection and analysis ensured consistency across all clinical sites. All images were evaluated using standard DXA manufacturer software. Cutlines were placed on the whole-body DXA scan to evaluate lean body mass and fat mass in four regions of interest (i) the whole body (excluding head), (ii) arms, (iii) legs and (iv) the appendicular skeleton (consisting of arms and legs as a single region of interest). Percent lean mass was calculated as whole-body lean body mass divided by whole-body soft-tissue mass (fat mass and lean body mass). The central reviewer was blinded to the study treatment assignment and other clinical information on the participant. To ensure DXA scanner consistency over time, quality assurance scans were acquired and the data were monitored centrally. Whole-body DXA scans were collected at baseline and at 16-week intervals throughout 96 weeks.

### Statistical analyses

A mixed model for repeated measures with terms for stratification factor, baseline result, treatment, time and treatment by time interaction as fixed effects and participants as random effect was used to assess the difference in DXA measurements between domagrozumab and placebo groups at Week 49. The %CFB for each visit was attributed to the last dose received at the previous visit. Baseline was defined as the last assessment collected prior to the first day of dosing.

The relationship between DXA lean body mass endpoints assessed at Week 49 and functional endpoints (4SC and NSAA) assessed at Week 97 was evaluated using simple linear regression and regression tree methods. For each pairwise comparison (DXA vs. functional endpoint), a regression tree was constructed to “split” the DXA endpoint into two subgroups that yielded the smallest level of variability on the functional endpoint within each subgroup. For these analyses, all participants from each treatment sequence were combined and only those participants with a Week 97 functional assessment were included. All *p* values are presented nominally without an adjustment for multiplicity, and statistical significance was assessed at the 0.05 level. The linear regression and regression tree methodology was designed to match MRI biomarker analysis in this same population^22^.

## Supplementary Information

Supplementary Information.

## Data Availability

Upon request, and subject to review, Pfizer will provide the data that support the findings of this study. Subject to certain criteria, conditions and exceptions, Pfizer may also provide access to the related individual de-identified participant data. See https://www.pfizer.com/science/clinical-trials/trial-data-and-results for more information.
