# Supplementary material for: Dual-energy X-ray absorptiometry measures of lean body mass as a biomarker for progression in boys with Duchenne muscular dystrophy
Source: Sci Rep. 2022 Nov 5;12:18762. doi: 10.1038/s41598-022-23072-5 (PMC9637094; doi:10.1038/s41598-022-23072-5)
Supplement: Supplementary file 1 — Supplementary Information. [file 41598_2022_23072_MOESM1_ESM.pdf]

Supplementary Table S1 List of addresses of institutional review boards or ethics committees for participating sites in the original clinical trial

Country	Addresses of institutional review boards or ethics committees
Australia	Children's Health Queensland Hospital and Health Service Human Research Ethics Committee<br>62 Graham Street, Lady Cilento Children's Hospital Precinct, Level 7, Centre for Children's Health Research<br>South Brisbane, QLD 4101
Bulgaria	ETHICS COMMITTEE FOR MULTICENTER TRIALS<br>5, Sveta Nedelya sq.<br>Sofia, 1000
Canada	Conjoint Health Research Ethics Board (CHREB, University of Calgary)<br>2500 University Drive NW, 3rd Floor Mackimmie Library Tower (MLT 300)<br>Calgary, AB T2N 1N4
	Western University Health Science Research Ethics Board<br>Support Services Building<br>1393 Western Rd, Room 5150<br>London, ON N6G 1G9
	UBC Children's and Women's Research Ethics Board<br>950 West 28th Avenue, Room A2-141A<br>Vancouver, BC V5Z 4H4
	IRB, CHU Sainte-Justine<br>3175 Cote-Sainte-Catherine<br>Montreal, QC H3T 1C5
Italy	Comitato Etico Fondazione Policlinico Universitario A. Gemelli<br>Largo Gemelli, 8<br>Rome, 00168
	Comitato Etico IRCCS Ospedale Pediatrico Bambino Gesu<br>Viale Ferdinando Baldelli, 41<br>Rome, 00146
	Comitato Etico Regione Liguria Sezione 3 c/o IRCCS Azienda Ospedaliera Universitaria San Martino-IST

	Largo Rosanna Benzi, 10<br>Genova, 16132
Japan	National Center of Neurology and Psychiatry Institutional Review Board<br>4-1-1 Ogawahigashi, Kodaira<br>Tokyo, 187-8551
	Hyogo College of Medicine Hospital Institutional Review Board<br>1-1 Mukogawacho, Nishinomiya-shi<br>Hyogo, 663-8501
Poland	Komisja Bioetyczna przy Warszawskim Uniwersytecie Medycznym<br>ul. Żwirki i Wigury 61<br>Warszawa, 02-091
United Kingdom	NRES Committee Yorkshire and the Humber – Leeds East<br>Jarrow Business Centre<br>Rolling Mill Road, Room 001<br>Jarrow, NE32 3DT
United States	Washington University in St. Louis, Human Research Protection Office<br>660 S Euclid Ave, Campus Box 8089<br>St. Louis, MO 63110
	WESTERN IRB<br>1019 39th Ave SE, Ste 120<br>Puyallup, WA 98374
	UCLA Medical Institutional Review Board #3 (MIRB3)<br>11000 Kinross Ave, Ste 211<br>Los Angeles, CA 90095
	Cincinnati Children's Institutional Review Board<br>3333 Burnet Ave, MLC 7040<br>Cincinnati, OH 45229
	University of Minnesota Institutional Review Board<br>D528 Mayo Memorial Bldg, 420 Delaware St SE

	Minneapolis, MN 55455
	Office of Research, IRB Administration<br>UC Davis Medical Center<br>2921 Stockton Blvd, Ste 1400<br>Sacramento, CA 95817
	Johns Hopkins Medicine Office of Human Subjects Research Institutional Review Boards<br>1620 McElderry St,Reed Hall, Ste B-130<br>Baltimore, MD 21205-1911
	Duke University Health System Institutional Review Board<br>2424 Erwin Rd, DUMC Box 2712, Ste 405 Hock Plaza<br>Durham, NC 27705
	University of Utah Institutional Review Board<br>75 S 2000 E, Research Administration Bldg<br>Salt Lake City, UT 84132
	The Children's Hospital of Philadelphia Institutional Review Board<br>2716 S St, The Roberts Center for Pediatric Research, 4th Fl<br>Philadelphia, PA 19146
	Partners Human Research Committee (PHRC)<br>399 Revolution Dr, Ste 710<br>Someville, MA 02145
	Partners Human Research Committee (PHRC)<br>116 Huntington Ave, Ste 1002<br>Boston, MA 02116
	Ann and Robert H. Lurie Children's Hospital of Chicago Institutional Review Board<br>225 E Chicago Ave<br>Chicago, IL 60611
	Human Subjects Committee,University of Kansas Medical Center<br>3901 Rainbow Blvd<br>Kansas City, KS 66160
